# Patient Safety Culture and Its Associated Factors: A Situational Analysis among Nurses in Katsina Public Hospitals, Northwest Nigeria

**DOI:** 10.3390/ijerph19063305

**Published:** 2022-03-11

**Authors:** Musa Sani Kaware, Mohd Ismail Ibrahim, Mohd Nazri Shafei, Suhaily Mohd Hairon, Abduljaleel Umar Abdullahi

**Affiliations:** 1 Department of Community Medicine, School of Medical Sciences, Universiti Sains Malaysia, Kubang Kerian 16150, Kelantan, Malaysia; musaskaware@student.usm.my (M.S.K.); drnazri@usm.my (M.N.S.); suhailymh@usm.my (S.M.H.); 2 Department of Community Medicine, College of Health Sciences, Umaru Musa Yar’adua University, Katsina 820101, Katsina State, Nigeria; abduljalil.abdullahi@gmail.com

**Keywords:** patient safety culture, situational analysis, nurses, public hospitals, medical errors

## Abstract

Background: Patient safety involves identifying, assessing, and managing patient-related risks and occurrences to improve patient care and reduce patient harm. In Nigeria, there is a lack of studies on patient safety culture, especially in the northern part of the country. This study aimed to determine the levels and factors that contribute to nurses’ negative perceptions of patient safety culture in public health facilities. Methodology: A total of 460 nurses were surveyed across 21 secondary health facilities using the Hospital Survey on Patient Safety Culture, and the response rate was 93.5%. Descriptive statistics and multiple logistic regression were used to analyze the data. Results: The results showed that 59.8% of the respondents were female, and 42.6% were within the age range of 30–39 years old. Most of them (48.3%) had spent 1–5 years working in the hospital. Three out of 12 composite measures had higher negative responses (staffing—30.5%, non-punitive response to error—42.8%, and frequency of events reported—43.1%). A multiple logistic regression analysis affirmed that all three variables, in addition to organizational learning, were significant associated with overall negative perceptions of patient safety culture, with 3.15, 1.84, 2.26, and 2.39 odds ratios, respectively. Conclusion: The results revealed that four critical areas of patient safety required improvement; therefore, intervention is recommended to minimize unnecessary patient harm and medical expenses.

## 1. Introduction

Safety culture is a term used to assess “the attitudes, beliefs, and perceptions shared by natural groups as defining norms and values” [1], which determine how they react concerning reporting, analyzing, and preventing errors that can develop into life-threatening circumstances or outcomes. This is linked to the concepts of assessing hazards, risk, harm, and the identification of errors, events, and incidents [2]. Research has been indicated that the main factors responsible for causing patient harm have been communication problems, staffing patterns, poor or lack of error reporting systems, organizational transfer of knowledge, inadequate information flow, individual problems, inadequate policies and procedures, and technical failures [3].

Some literature from high-income countries has shown that a significant number of patients are being harmed in the process of healthcare, leading to either increased cost of medical care, extended time of stay in the healthcare facilities, permanent disabilities, or even death [4]. Recent studies have revealed that medical errors are the third leading cause of death in the United States of America after cancer and heart disease [3]. Another study has also divulged that, on average, every 35 s, at least one case of patient harm is reported in the United Kingdom [4,5]. In addition, studies conducted in American states, such as Colorado, Utah, and New York have revealed that no less than 44,000 and as many as 980,000 American people die in hospitals as a result of preventable medical errors, such as medication, surgical, and diagnostic errors, every year [6]. The reports further ascertained that even if the lowest estimate was considered, it surpassed the number of deaths ascribed to vehicle accidents, breast cancer, and AIDS [6]. Comparatively, in low- and middle-income countries, a combination of undesirable factors, such as understaffing, inadequate infrastructure, poor hygiene and sanitation, overcrowding, lack of healthcare commodities, and shortage of essential equipment, has contributed enormously to the serious condition of patient safety [4,7].

It is further estimated that the overall annual frequency of hospitalization has reached up to 421 million worldwide, of which 42.7 million adverse events occur in hospitalized patients [4,8]. It has also been stated that low- and middle-income countries account for about two-thirds of all adverse events globally [9]. In a report titled Patient Safety: Making Healthcare Safer, the World Health Organization (WHO) stated that: “Treating and caring for patients in a safe environment and protecting them from healthcare-related avoidable harm should be a national and international priority and called for concerted international efforts” [4]. In addition, in 2004, the World Alliance for Patient Safety and the WHO called for attention in sub-Saharan African countries for urgent understanding, action, and improvement of patient safety culture [8,10].

The lack of sufficient data on incidence reporting among sub-Saharan African countries has made it difficult to measure the intensity of adverse events and has made the region an environment of preventable morbidity and mortality due to poor infection control practices and overcrowding in hospitals [11]. According to the WHO regional director for Africa, the majority of the countries in the region do not have a national policy on safe healthcare practices [12]. However, more local organizations have recently emerged in the region with the goal of developing measures to enhance patient care through accreditation efforts connected to the Joint Commission International and the Council for Health Service Accreditation of Southern Africa [13]. An example of such an organization in sub-Saharan Africa is the Society for Quality Healthcare in Nigeria (SQHN). The SQHN was formed with a mission to lead, advocate, and facilitate the continuous improvement of quality and safety in healthcare [13,14]. Despite the calls by the WHO and other health organizations on improving patient safety culture in the region, there is still not much research on patient safety culture in Nigeria, particularly in the northern part of the country.

In Nigeria, poor patient safety practices in public healthcare facilities have become a significant public health challenge due to one or a combination of factors related to healthcare provider- or patient-related factors. The most common patient safety challenges in Nigeria include, but are not limited to, surgery, medication, diagnostics, transfusion, healthcare-associated infection, staff competency, emergency management, medical equipment, communication, accessibility, reduced error reporting, and management systems [9]. Thus, it has become necessary to carry out a baseline assessment of the patient safety culture to determine the level and associated factors in the region and to identify the areas that need intervention.

## 2. Materials and Methods

### 2.1. Setting and Study Design

This research is a cross-sectional study using the Hospital Survey on Patient Safety Culture (HSOPSC) developed by the Agency for Health Research and Quality (AHRQ) to assess patient safety culture among nurses in Katsina State public hospitals in northwestern Nigeria. The study was conducted among nurses in 20 public secondary health facilities across the state.

### 2.2. Study Area

Nigeria’s government is divided into three levels: federal, state, and local. The obligation to deliver healthcare in the public sector is shared among the three levels of government. Clinics, dispensaries, and health posts act as the community’s entrance point into the local government’s healthcare system, providing general preventative, preventive, promotive, and pre-referral therapy [15,16]. These facilities are generally staffed with nurses, environmental health officers, community health workers, community health extension workers (CHEWs), and junior CHEWs. Patients referred from primary healthcare are admitted to secondary facilities that provide general medical and laboratory services, as well as specialty health services such as surgery, pediatrics, obstetrics, and gynecology. General hospitals often employ medical officers, nurses, midwives, laboratory and pharmacy professionals, and community health officials. Tertiary-level facilities, which include specialty and teaching hospitals as well as federal medical centers (FMCs), are the highest level of healthcare in Nigeria. They handle patients referred from the primary and secondary levels and have specialized expertise and complete technological capability, allowing them to serve as knowledge-generating and dissemination resource centers. At least one tertiary institution exists in each state [13,17,18,19].

### 2.3. Sampling Method

Before data collection commenced, an introduction letter was obtained from the Katsina State Hospital Management Board (KSHMB). In addition, in each of the 20 secondary health facilities, a meeting was conducted with the medical director and head of nursing services to formally introduce the research and its procedure, purposes, and benefits, as well as to seek their support for the successful conduct of the study.

The sample size for the study was calculated according to the study objectives using single-proportion formula and PS Power and Sample Size Calculation software version 3.1.2 by William D. Dupont and Walton D. Plummer, Vanderbilt University, Nashville, TN, USA. The highest value obtained (460) was therefore used as the study sample size. To obtain the required sample size, a compiled list of available staff was obtained from the head of the nursing service in each hospital for a simple random selection of respondents. In each of the study hospitals, one research assistant was assigned to facilitate the collection of completed surveys.

The survey instrument for this study was a paper-format self-administered questionnaire (HSOPSC), which took the respondents 10–15 min to complete. This format was chosen because of its feasibility for most public hospitals in Nigeria, and the AHRQ encouraged the use of a paper format for the highest possible response rate [20].

### 2.4. Research Tool

The research tool used in this study was the Hospital Survey on Patient Safety Culture (HSOPSC), which was developed by the AHRQ [21]. The agency has been continually using this survey instrument in hospitals in the United States to compile data for its database and publish annual reports on the status of patient safety culture since it was developed [21].

In addition, several researchers have reported the applicability of this questionnaire in healthcare settings from different countries around the globe, including Jordan [22], Sweden [23], Egypt [24], Afghanistan [25], Saudi Arabia [26,27,28], Slovenia [29], the Netherlands [30], Lebanon [31], Iran [32], Taiwan [33], Kuwait [34], Brazil and Portugal [35], Switzerland [36], and many other countries. The current survey does not require any translation or validation, with English being the official language in Nigeria.

The HSOPSC contains 42 items, which are further grouped into 12 composite measures: teamwork within units or departments; supervisor or manager expectations and actions promoting patient safety; organizational continuous learning improvement; hospital management support for patient safety; overall perceptions of patient safety; feedback and communication about errors; communication openness; frequency of events reported; teamwork across hospital units; staffing; hospital handoffs and transitions; and non-punitive responses to errors. In addition, the respondents were requested to provide their background information, such as age, gender, duration of work experience in the current unit and hospital, and whether they had direct contact or interaction with patients, among others [20].

The scaling of the survey instrument is based on a 5-point Likert scale as either strongly disagree, disagree, neutral, agree, or strongly agree. Some of the survey’s composites were rated as either never, rarely, sometimes, most of the time, or always. Both rating scales were coded with score numbers (1, 2, 3, 4, and 5, respectively) for easy data entry and analysis.

The AHRQ’s HSOPSC was pilot tested among 1437 respondents in 21 hospitals across six states in the United States. The factor structure and reliability of the survey composites were examined and analyzed, and they were confirmed to be psychometrically sound. The results provided overall evidence supporting the 12 dimensions and 42 items included in the HSOPSC as having acceptable psychometric properties at all levels of analysis. Cronbach’s alpha for the composites ranged from 0.62 to 0.85, with an average of 0.77. All composites had acceptable reliability (0.70 or higher), except for the staffing composite (α = 0.62) [37]. The lower reliability of the survey tool can be attributed to differences in the respondents’ demographic characteristics and their levels of heterogeneity. In addition, the lack of modification and inconsistencies in the assessments of constructive validity were additional factors to explain the differences [37,38,39,40].

### 2.5. Data Collection

The HSOPSC was distributed to one point of contact in the various units or departments that were accessible to the respondents at the beginning of their working days in each hospital. The distribution of the survey was accompanied by a supporting cover letter guiding the respondents on how to complete and return the survey, and a consent form. Furthermore, the cover letter requested that the respondents complete the survey within three days, even though the deadline was not specified in the cover letter, because data collection might have been delayed or rescheduled [27].

To ensure uniformity, easy tracking of non-respondents, and redistribution of the survey, each survey tool was given a unique ID-tracking code. This code was recorded on a tracking log sheet that was given to the research assistant in each hospital. Moreover, this tracking log sheet was used to trace the unreturned surveys and other staff members who might not have received the survey. The tracking log sheet carried only the survey tracking number but no other identity of the respondents to ensure their anonymity. The entire data collection activity was completed in six weeks.

### 2.6. Data Processing and Analysis

A total of 460 registered nurses were surveyed from secondary health facilities (general hospitals), of which 434 responded to the survey tool, making the response rate 93.5%. Four of the responded surveys were invalid and excluded from the analysis. The data were analyzed using SPSS version 24, Armonk, NY, USA, IBM Corp, Statistical Package for the Social Sciences. Before calculating the percentage of positive and negative scores, missing responses were identified and excluded, and negatively worded questions were reversed. The top two response categories (strongly agree and agree, or most of the time and always) were merged and considered positive responses. The remaining three response categories (strongly disagree, disagree, and neither or never, rarely, and sometimes) were merged and considered negative responses for the purpose of statistical analysis.

Descriptive statistics (percentage and frequency) were used to describe the background and job-related characteristics of the respondents and the level of patient safety culture in the hospitals. A *p*-value of 0.05 was used as the statistical significance level. Multiple logistic regression analyses were used to determine the association between the dependent and independent variables. According to the HSOPSC user guide, there are 12 composites involved in the questionnaire, each of which is independent and mutually exclusive. We decided to take the overall perceptions of the patient safety culture composite as a dependent variable, while the others, including the sociodemographic data, were used as independent variables. The scores for each item were grouped into positive and negative to obtain a binary outcome variable based on the HSOPSC guidelines [20]. The regression analysis was performed using the stepwise backward option for all independent variables separately. The odds ratio with a 95% confidence interval was examined to determine the factors associated with overall negative perceptions of patient safety culture.

## 3. Results

Table 1 shows the background and job-related characteristics of the subjects, which were divided into different variables. Of the total number of nurses (430) who participated in the study, 257 (59.8%) were female, and the remaining 173 (40.2%) were male. In addition, most of the respondents in the study (42.6%) were within the age range of 30–39 years, while the smallest percentage (3.0%) were aged between 60 and 69 years old. With regard to the years of experience in the hospital, most of the respondents (48.3%) have spent 1–5 years working in the hospital, followed by those who have worked there 6–10 years (20.4%), whereas 2.1% have been working in the hospital for 16–20 years.

Among the 430 nurses participating in the study, 52.4% worked in their current units for 1–5 years, followed by those who worked in their current units for less than 1 year (32.0%). Similarly, 46.4% of the respondents reported that they worked 40–59 h per week, whereas only 3.6% of the respondents said they worked less than 20 h per week. The findings of this study further revealed that the majority of the nurses (40.5%) had only worked in the profession for 1–5 years, while only 13.2% of the respondents had been in the nursing profession for over 20 years. Moreover, it was noticed from the results that about 96.9% of the study participants had direct contact with patients, and only 3.1% did not have direct contact with patients.

Table 2 displays the percentages of the respondents answering negatively or positively to the survey items. It shows that out of the 12 composites of patient safety culture, staffing had the highest negative responses (69.5%), followed by non-punitive response to error (57.2%) and frequency of event reporting (56.9%). On the other hand, teamwork within units scored a higher percentage of positive responses (91.1%), followed by organizational learning and continuous improvement of patient safety culture (84.7%), teamwork across units (83.0%), and management support for patient safety (80.3%).

Similarly, the table also displays the item with the highest negative perception: “Staff in this unit work longer hours than is best for patient care” (85.3%); then “We have enough staff to handle the workload” (78.9%); followed by “When a mistake is made but has no potential to harm the patient, how often is this reported?” (69.4%); “Staff worry that mistakes they make are kept in their personnel file” (67.8%); “We work in ‘crisis mode’ trying to do too much, too quickly” (67.2%); “Staff feel like their mistakes are held against them” (56.0%); and “It is just by chance that more serious mistakes don’t happen around here” (55.4%).

For the positive perception, the survey item having the highest score was “We are actively doing things to improve patient safety” (97.9%); then “In this unit, people treat each other with respect” (95.8%); followed by “When a lot of work needs to be done quickly, we work together as a team to get the work done” (94.4%); “My supervisor or manager seriously considers staff suggestions for improving patient safety” (92.4%); “After we make changes to improve patient safety, we evaluate their effectiveness” (91.7%); “My supervisor or manager says a good word when he or she sees a job done according to established patient safety procedures” (91.3%); and “Hospital units work well together to provide the best care for patients” (91.2%).

Table 3 presents the results for simple and multiple logistic regression analyses to determine the odds ratio of possible risk factors associated with an overall negative perception of patient safety culture. From the simple logistic regression performed, a total of 16 variables had *p*-values of <0.25. On the basis of a study by Bursac et al. (2008), it was noted that using a cutoff point of 0.05 can fail to identify variables known to be important [41]. Hence, the variables with a *p*-value < 0.25 were included in the multiple logistic regression analysis. Four variables were retained in the final model, and they were included using the enter method to obtain the preliminary main effect model. Overall negative perceptions of patient safety culture was used as a dependent variable. The results in Table 3 show that there are four factors that are significantly associated with staff’s overall negative perceptions of patient safety culture. Staff who had reported fewer events (five or less) are more likely to have overall negative perceptions of patient safety culture than those who had reported more than five, with an odds ratio of 2.66 (95% CI = 1.03–4.97). Nurses who had negative perceptions of organizational learning and continuous improvement, negative perceptions of staffing, and negative perceptions of handoffs and transition were significantly associated with overall negative perceptions of patient safety culture, with adjusted odds ratios of 2.39 (95% CI = 1.40–4.10), 3.15 (95% CI = 1.34–7.17), and 1.48 (95% CI = 1.09–3.12), respectively.

## 4. Discussion

To the best of our knowledge, this study is the first of its kind to be conducted in northwestern Nigeria. In this study, we tried to assess the level of patient safety culture and its associated factors among nurses in certain public health facilities. The findings showed that a majority of the survey composites scored more than the average level of positive perceptions and are referred to as areas of strength. These include teamwork within a unit; organizational learning and continuous improvement; teamwork within units; management support for patient safety; supervisor’s expectations and actions promoting patient safety, feedback, and communication about errors; handoffs and transitions; communication openness; and overall perceptions of patient safety. The only three composites that scored below average were staffing, non-punitive response to error, and frequency of events reported.

Most of the nurses reported that they work longer hours than is good for the patient, while some mentioned that they do not have enough staff to handle the workload. Staffing is one of the key aspects in the quality of healthcare services and patient care outcomes. Whenever there is poor staffing in a hospital, many areas of service delivery cannot work effectively. However, the issue of understaffing in healthcare facilities is a global problem, as many studies have also revealed evidence of poor staffing in many countries. These include research conducted on patient safety culture and associated factors in hospitals in the Jima zone in southwest Ethiopia [42], Saudi Arabi [43], Lebanon [31], Sweden, Spain, Hungary, and Croatia [44]. This finding was further attested by a logistic regression analysis in the present study, which showed that an increase in the negative perception of staffing composite has the odds of 3.15 to a negative perception of the overall patient safety culture.

Nonpunitive response to error is another weak dimension that requires serious attention. It is all about how staff feel that when they make mistakes or report an event, it will be held against them, and the mistakes will be reported in their personnel files. The overall score of the average positive response to non-punitive response to error in this study is 42.8%. This indicated that the nurses in the studied hospitals feared being blamed when they made mistakes instead of correcting them. Moreover, they showed their fears that when they made mistakes, they would be kept in their files. The consequences of this fear may result in continuously occurring medical errors in hospitals without identifying and correcting them. Thus, it is of paramount importance for hospital management to create an avenue where healthcare workers at liberty to report errors and contribute to ways of minimizing them. However, in comparison with previous studies [26,30,32,33], the present findings show a better result. In addition, a study conducted to assess patient safety culture in hospital settings by Abdulmajeed et al. (2021) identified error reporting as one of the factors that required improvement [45]. However, the present findings showed a better result, which is even closer to the AHRQ benchmark of 43% [33].

The dimension of frequency of error reporting measured the rate of occurrence of medical events or mistakes that have the potential to harm patients directly or indirectly. In some instances, the error can occur and be corrected before affecting the patient, and sometimes it may happen and cause serious injury or even death to the patient.

In the present study, most of the nurses expressed negative perceptions of the frequency of error reporting. A majority of them said that when a mistake is made but has no potential to harm the patient, they do not report it, or they rarely report it. Meanwhile, half of them said that they did not normally report errors that were caught and corrected before affecting the patient. This indicates that many errors are happening daily in the hospitals, and that there is a tendency to not report even the errors that have the potential of harming the patients, which may be the result of understaffing, fear of punishment, or lack of error reporting systems. This failure of error or adverse event reporting made it difficult to understand the true number of errors, the types of errors, and the magnitude of harm to patients. This study is in agreement with the findings of a study conducted on the assessment of patient safety culture among healthcare providers at Ain Shams University hospitals in Cairo, Egypt [46], which showed that the average positive response score for error reporting is 33.4%. However, it is also inconsistent with the results of an evaluation of patient safety culture in a secondary care setting in Kuwait conducted by Alqattan in 2017 [47].

Another dimension identified as an important determinant of patient safety culture in this study is hospital staff handoffs and transitions. Handoff is the process that deals with the transfer of essential patient information during shift changes between healthcare providers or from one hospital unit to another to ensure the continuity of patient care [48]. Even though the dimension received a high positivity score, the logistic regression analysis identified it as a significant predictor of patient safety culture. As presented in the results section, we realized that a negative perception of handoffs and transition would increase a likely negative perception of the overall perception of patient safety culture by the odds of 1.48, when compared with a positive perception. This can be attributed to the lack of enough nursing staff to take care of the patients, which means that they are too busy to carry out a formal handover in their units and departments of work. In addition, time constraints may be another factor that can lead to a poor perception of handoffs and transitions. The results of similar research conducted in primary healthcare units in Turkey [49] and in a Saudi Arabian hospital [50] were consistent with the present study.

### Limitations of the Study

This study was based on nurses’ experience only, and thus it did not cover all the healthcare professional groups working in Katsina State secondary health facilities. In addition, primary, tertiary, and private health facilities were not covered, which makes it difficult to generalize the perceived overall patient safety culture results among healthcare providers. However, despite these limitations, this study has provided baseline data on the current situation of patient safety culture among nurses in public secondary health facilities. It also identified areas of weakness that require further improvement for better patient care.

## 5. Conclusions

This study examined the level of patient safety culture and its associated factors among nurses. The findings revealed that the majority of the survey composites scored positively above average. However, there are four critical areas of patient safety culture that require improvement: organizational learning, staffing, handoffs and transitions, and frequency of event reporting. Thus, it is recommended that all stakeholders in hospital management and policy makers establish a voluntary and mandatory error reporting system that will focus on identifying all sorts of errors or mistakes that may affect the quality of patient care in hospitals. In addition, similar research is recommended that will cover both public and private, primary, secondary, and tertiary health facilities across the region among all the professional groups in the healthcare system. Furthermore, an intervention is recommended to improve nurses’ knowledge of medical error reporting, its importance, and the possible consequences attached to it. For future research, a larger sample size should be used to cover all professional groups in the health service system.

## Figures and Tables

**Table 1:** Background and job-related characteristics of the respondents (*n* = 430).

Variable		Frequency (*n*)	Percent (%)
Gender	Male	173	40.2
	Female	257	59.8
Age group (year)	20–29	93	21.6
	30–39	183	42.6
	40–49	80	18.6
	50–59	61	14.2
	60–69	13	3.0
Duration of work experience in the hospital (year)	<1	47	11.1
	1–5	204	48.3
	6–10	86	20.4
	11–15	49	11.6
	16–20	9	2.1
	≥21	27	6.4
Duration of work experience in the current unit (year)	<1	135	32.0
	1–5	221	52.4
	6–10	40	9.5
	11–15	16	3.8
	16–20	5	1.2
	≥21	5	1.2
Number of working hours per week	<20	15	3.6
	20–39	117	28.3
	40–59	192	46.4
	60–79	43	10.4
	80–99	23	5.6
	≥100	24	5.8
Duration of work experience in the profession (year)	<1	39	9.4
	1–5	169	40.5
	6–10	79	18.9
	11–15	56	13.4
	16–20	19	4.6
	≥21	55	13.2
Direct contact with the patients	Yes	408	96.9
	No	13	3.1
Number of events reported in the past 12 months	0	220	56.7
	1–2	86	22.2
	3–5	44	11.3
	6–10	18	4.6
	11–20	10	2.6
	≥21	10	2.6
An overall grade on patient safety for the current unit.	Excellent	91	22.6
	Very good	205	51.0
	Acceptable	102	25.4
	Poor	4	1.0

**Table 2:** Summary of the percentage of negative and positive responses to patient safety culture by composites and items (*n* = 430).

Composites and Items		Negative Responses	Positive Responses
		*n* (%)	*n* (%)
**Teamwork within Units**		**151 (8.9)**	**1550 (91.1)**
a1.	People support one another in this unit.	20 (4.8)	399 (95.2)
a3.	When a lot of work needs to be done quickly, we work together as a team to get the work done.	24 (5.6)	404 (94.4)
a4.	In this unit, people treat each other with respect.	18 (4.2)	411 (95.8)
a11.	When one area in this unit gets really busy, others help out.	89 (20.9)	336 (79.1)
**Supervisor’s or Manager’s Expectations and Actions Promoting Patient Safety**		**422 (25.2)**	**1253 (74.8)**
b1.	My supervisor or manager says a good word when he or she sees a job done according to established patient safety procedures.	37 (8.8)	386 (91.3)
b2.	My supervisor or manager seriously considers staff suggestions for improving patient safety.	32 (7.6)	391 (92.4)
b3r.	Whenever pressure builds, my supervisor or manager wants us to work faster, even if it means taking shortcuts.	183 (44.1)	232 (55.9)
b4r.	My supervisor or manager overlooks patient safety problems that happen repeatedly.	170 (41.1)	244 (58.9)
**Organizational Learning—Continuous Improvement**		**194 (15.3)**	**1072 (84.7)**
a6.	We are actively doing things to improve patient safety.	9 (2.1)	419 (97.9)
a9.	Mistakes have led to positive changes here.	150 (36.1)	265 (63.9)
a13.	After we make changes to improve patient safety, we evaluate their effectiveness.	35 (8.3)	388 (91.7)
**Management Support for Patient Safety**		**249 (19.7)**	**1012 (80.3)**
f1.	Hospital management provides a work climate that promotes patient safety.	57 (13.3)	371 (86.7)
f8.	The actions of hospital management show that patient safety is a top priority.	64 (15.4)	353 (84.7)
f9r.	Hospital management seems interested in patients’ safety only after an adverse event happens.	128 (30.8)	288 (69.2)
**Overall Perceptions of Patient Safety**		**681 (41.3)**	**968 (58.7)**
a10r.	It is just by chance that more serious mistakes don’t happen around here.	226 (55.4)	182 (44.6)
a15.	Patient safety is never sacrificed to get more work done.	202 (50.5)	198 (49.5)
a17r.	We have patient safety problems in this unit.	206 (49.3)	212 (50.7)
a18.	Our procedures and systems are good at preventing errors from happening.	47 (11.1)	376 (88.9)
**Feedback and Communication About Error**		**355 (27.8)**	**920 (72.2)**
c1.	We are given feedback about changes put into place based on event reports.	172 (40.8)	250 (59.2)
c3.	We are informed about errors that happen in this unit.	110 (25.9)	315 (74.1)
c5.	In this unit, we discuss ways to prevent errors from happening again.	73 (17.1)	355 (82.9)
**Communication Openness**		**385 (30.2)**	**888 (69.8)**
c2.	Staff will freely speak up if they see something that may negatively affect patient care.	66 (15.5)	361 (84.5)
c4.	Staff feel free to question the decisions or actions of those with more authority.	207 (49.3)	213 (50.7)
c6r.	Staff are afraid to ask questions when something does not seem right.	112 (26.3)	314 (73.7)
**Frequency of Events Reported within last 12 months**		**712 (56.9)**	**539 (43.1)**
d1.	When a mistake is made but is caught and corrected before affecting the patient, how often is this reported?	219 (52.3)	200 (47.7)
d2.	When a mistake is made but has no potential to harm the patient, how often is this reported?	290 (69.4)	128 (30.6)
d3.	When a mistake is made that could harm the patient, but does not, how often is this reported?	203 (49.0)	211 (51.0)
**Teamwork Across Units**		**286 (17.0)**	**1397 (83.0)**
f2r.	Hospital units do not coordinate well with each other.	78 (18.3)	349 (81.7)
f4.	There is good cooperation among hospital units that need to work together.	40 (9.4)	386 (90.6)
f6r.	It is often unpleasant to work with staff from other hospital units.	131 (32.0)	279 (68.1)
f10.	Hospital units work well together to provide the best care for patients.	37 (8.8)	383 (91.2)
**Dimensions and Items**		**Negative Response**	**Positive Response**
		***n* (%)**	***n* (%)**
**Staffing**		**1156 (69.5)**	**508 (30.5)**
a2r.	We have enough staff to handle the workload	336 (78.9)	90 (21.1)
a5r.	Staff in this unit work longer hours than is best for patient care	359 (85.3)	62 (14.7)
a7r.	We use more agency/temporary staff than is best for patient care	191 (46.0)	224 (54.0)
a14r.	We work in “crisis mode” trying to do too much, too quickly	270 (67.2)	132 (32.8)
**Handoffs and Transitions**		**478 (28.6)**	**1196 (71.4)**
f3r.	Things “fall between the cracks” when transferring patients from one unit to another	132 (32.0)	281 (68.0)
f5r.	Important patient care information is often lost during shift changes	85 (20.1)	337 (79.9)
f7r.	Problems often occur in the exchange of information across hospital units	191 (45.7)	227 (54.3)
f11r.	Shift changes are problematic for patients in this hospital	70 (16.6)	351 (83.4)
**Nonpunitive Response to Errors**		**696 (57.2)**	**520 (42.8)**
a8r.	Staff feel like their mistakes are held against them	228 (56.0)	179 (44.0)
a12r.	When an event is reported, it feels like the person is being written up, not the problem	184 (47.2)	206 (52.8)
a16r.	Staff worry that mistakes they make are kept in their personnel file	284 (67.8)	135 (32.2)

Keys: r = reversed question. *n* = number of responses. Positive responses = sum of agree and strongly agree responses. Negative responses = sum of disagree, strongly disagree, and neither response.

**Table 3:** Logistic regression analysis to determine the factors associated with negative perceptions of patient safety culture (*n* = 430).

Variable	Categories	Simple Logistic Regression		Multiple Logistic Regression		
		COR (95% CI)	*p*-Value	AOR (95% CI)	Wald Stat (df)	*p*-Value
Age	<40 years old	1				
	≥40 years old	0.87 (0.58–1.29)	0.479			
Gender	Male	1				
	Female	0.84 (0.57–1.23)	0.371			
Years of experience in the hospital	<5 years	1				
	≥5 years	0.63 (0.39–1.03)	0.063			
Years of experience in the current unit	<5 years	1				
	≥5 years	0.56 (0.24–1.28)	0.168			
Working hours per week	<40 h	1				
	≥40 h	1.14 (0.75–1.72)	0.546			
Years of experience in the profession	<5 years	1				
	≥5 years	0.63 (0.41–0.96)	0.033			
Number of events reported in last 12 months	High	1		**1**		
	Low	1.78 (0.90–3.53)	0.098	**2.26 (1.03–4.97)**	**4.109**	**0.043**
Direct contact with patients	No	2.52 (1.01–6.31)	0.048			
	Yes	1				
Teamwork within units	Positive	1				
	Negative	2.22 (0.95–5.18)	0.066			
Supervisor’s expectations and actions promoting patient safety	Positive	1		**1**		
	Negative	2.83 (1.74–4.62)	<0.001	**2.39 (1.40–4.10)**	**10.139**	**0.001**
Organizational learning continuous improvement	Positive	1				
	Negative	1.70 (1.06–2.73)	0.029			
Management support for patient safety	Positive	1				
	Negative	2.07 (1.31–3.27)	0.002			
Feedback and communication about error	Positive	1				
	Negative	2.00 (1.24–3.22)	0.005			
Communication openness	Positive	1				
	Negative	1.65 (1.07–2.55)	0.023			
Frequency of events reported	Positive	1				
	Negative	1.30 (0.88–1.91)	0.195			
Teamwork across nits	Positive	1				
	Negative	1.57 (0.90–2.75)	0.111			
Staffing	Positive	1		**1**		
	Negative	3.25 (1.62–6.51)	0.001	**3.15 (1.39–7.17)**	**7.492**	**0.006**
Handoffs and transitions	Positive	1		**1**		
	Negative	1.85 (1.18–2.90)	0.007	**1.84 (1.09–3.12)**	**5.159**	**0.023**
Nonpunitive response to errors	Positive	1				
	Negative	1.60 (1.04–2.46)	0.034			

Key notes: COR = crude odds ratio. AOR = adjusted odds ratio. CI = confidence interval. Variables with a *p*-value < 0.25 were included in the multiple logistic regression [41]. Forward or backward LR method used, no multicollinearity and no interaction, area under the curve 69.6%, classification table 65.6%, Hosmer–Lemeshow, *p* = 0.167. High = 6 or more events reported. Low ≤ 5 events reported.

## Data Availability

The authors can make the raw data from this study available to interested scholars upon request.
